# Positive short term effects of an integrative korean medicine treatment package for low back pain caused by motor vehicle accidents: A retrospective chart review of real – world practice data

**DOI:** 10.3389/fphar.2022.1003849

**Published:** 2022-10-17

**Authors:** Hye-Rin Park, Seungmin Kathy Lee, Sang-Hoon Yoon, Hee-Geun Jo, Ji-Yong Kim, Hyunho Kim, Jae-Uk Sul, Jungtae Leem

**Affiliations:** ^1^ Chung-Yeon Korean Medicine Hospital, Gwangju, South Korea; ^2^ Virginia University of Integrative Medicine, Fairfax, VA, United States; ^3^ QualTEAM Academy, Seoul, South Korea; ^4^ Chung-Yeon Korean Medicine Clinic, Seoul, South Korea; ^5^ Department of Herbal Pharmacology, College of Korean Medicine, Gachon University, Seongnam, South Korea; ^6^ Jayoenanae Korean Medicine Clinic, Busan, South Korea; ^7^ Department of Korean Rehabilitation Medicine, Dongshin University Korean Medicine Hospital, Gwangju, South Korea; ^8^ College of Korean Medicine, Wonkwang University, Iksan, South Korea

**Keywords:** integrative medicine, Korean medicine, acupuncture, herbal medicine, low back pain, motor vehicle accident, real world data, botanical drug

## Abstract

**Backgrounds** No standard treatment exist for reducing symptoms related to sequelae of motor vehicle accidents (MVAs). In Korea, comprehensive Korean Medicine (KM) treatment that includes botanical drugs (herbal medicine), acupuncture, pharmacopuncture, tuina, moxibustion, and cupping is covered by automobile insurance and increasingly used to help alleviate such pain. This study aimed to analyze real-world data and to evaluate the effectiveness and safety of comprehensive KM treatment for low back pain caused by MVAs.

**Methods** We conducted a retrospective chart review of patients who received KM treatment during hospitalization. Records that lacked follow-up outcome assessments were excluded. The Verbal Numerical Rating Scale (VNRS), the Korean version of the Oswestry Disability Index (K-ODI) and the Korean version of the Roland-Morris Disability Questionnaire (K-RMDQ) were evaluated at admission and discharge. Adverse events were also analyzed. A paired *t*-test was used to identify the effectiveness of KM treatment.

**Results** A total of 50 patients, 30 males and 20 females, were included in the analysis. The mean age of the patients was 40.72 ± 13.31 years and the average treatment period was 7.22 ± 3.84 days. After treatment, VNRS, K-ODI and K-RMDQ were significantly improved (*p* < 0.001). There was a decrease from 5.06 ± 1.60 to 3.40 ± 1.81 in VNRS, 33.38 ± 16.88 to 24.54 ± 13.63 in K-ODI, and 6.84 ± 6.27 to 4.14 ± 4.38 in K-RMDQ. During this period, a total of two adverse events were reported.

**Discussion** Although this retrospective chart review looked into the short term effects only, comprehensive KM treatment might be an effective and safe therapeutic option to reduce acute low back pain especially after MVA. Prospective research data is needed to support this hypothesis.

## Introduction

Motor vehicle accidents (MVAs) are predicted to become the third leading cause of disease burden in the world by 2020. (Murray, 1996). According to a 2018 global status report on road safety published from the World Health Organization (WHO), the number of deaths from road traffic accidents around the world is growing annually and tens of millions of people are known to suffer injuries and disabilities each year. (World Health Organization, 2018). As a result, the importance of managing sequelae of motor vehicle accident is also increasing worldwide.

Although MVAs can cause a variety of musculoskeletal pain, (Hincapié et al., 2010), many of these are not acute injuries requiring immediate medical treatment or hospitalization, and are often classified as “musculoskeletal sprains”. (Platts-Mills et al., 2012; Bortsov et al., 2013). However, low back pain (LBP) due to MVAs was a complaint in about 50% of the MVA patients who visited the initial medical institution, (Cassidy et al., 2003), and more than moderate LBP was found as commonly as neck pain at 6 weeks after the MVA. (Bortsov et al., 2014).

In Korea, according to the statistical analysis of traffic accidents published in 2018, among the major injuries caused by domestic traffic accidents, the low back region accounted for the highest percentage of injuries following the neck and other unspecified parts. (Kimho and Sung, 2018). LBP patients due to MVAs can suffer relatively more severe sequelae in daily life than general LBP patients. Having a history of LBP after MVAs can be a risk factor for future recurrent LBP. Thus, active treatment and management is important. (Jo et al., 2003; Nolet et al., 2018).

Conventional treatment methods for LBP after MVAs mainly include medication such as acetaminophen, non-steroidal anti-inflammatory drugs (NSAIDs), and muscle relaxants. (Wong et al., 2017). Opioids and benzodiazepines are also used for patients with severe pain and mental health disorders. (Berecki-Gisolf et al., 2016; Wong et al., 2017). However, NSAIDs may cause gastrointestinal side effects such as abdominal pain, diarrhea, and gastrointestinal bleeding, and muscle relaxants may cause symptoms such as drowsiness and dizziness. (Qaseem et al., 2017). In addition, opioids and benzodiazepines may have side effects such as drug abuse or addiction during long-term use. (Qaseem et al., 2017).

Therefore, alternative treatment strategies with fewer side effects and long-term applicability are needed to manage pain more effectively. In Korea, Korean Medicine (KM) treatment including botanical drugs (herbal medicine), acupuncture, pharmacopuncture, tuina, moxibustion, and cupping therapy, has been covered by automobile insurance since 1999 and such treatment modalities have been widely used to manage traffic accident patients in the clinic. According to automobile insurance medical expenses statistics presented by the Health Insurance Review and Assessment Service in Korea, the total cost of automobile insurance has increased over the years, (Health Insurance Review & Assessment Service, 2019), and among them, the usage of KM treatment has increased considerably compared to conventional medicine. (National Development Institute of Korean Medicine, 2016). Previous studies also report that the effectiveness and satisfaction of receiving KM treatment for sequelae of MVA are high among patients. (Park et al., 2015; Han et al., 2018; Kim et al., 2018; Shin et al., 2018). However, most of the post-MVA pain studies were limited to whiplash injuries, i.e. neck pain, (Bortsov et al., 2014), and there were relatively few studies looking into the effectiveness of KM treatment for acute LBP. Therefore, the purpose of this study was to evaluate the effectiveness and safety of comprehensive KM treatment for alleviating acute LBP after MVAs based on real-world practice data through a retrospective chart review of 50 patients.

## Patients and methods

### Selection of Patients

20-70-year-old male and female MVA patients with acute LBP were investigated from 1 July 2018 to 30 September 2018. All patients were admitted into a KM based hospital. Patients were excluded if the clinical outcome were not assessed at discharge, if the presenting symptoms were due to reasons other than simple muscle strain (eg. fracture of the lumbar vertebra) or if the patients were prescribed botanical drugs (herbal medicine) other than Chung-Pung Decoction, which is an botanical drugs frequently used for acute LBP treatment, during their hospitalization period. To reduce bias, this study did not allow inclusion of patient data that was treated using a different KM treatment protocol other than the methods stated below.

### Outcome measurements

We selected clinical outcome variables related to acute low back pain among which were evaluated at admission and discharge.

1. Verbal Numerical Rating Scale (VNRS)

The Verbal Numerical Rating Scale (VNRS) is a method for patients to express their pain intensity as a number between 0 and 10 verbally, assuming that the numbers 0 and 10 are ‘no pain’ and ‘the most or worst pain’ respectively. (Tsze et al., 2018). The Minimally Clinically Important Difference (MCID) of the NRS for low back pain is 1.5 points at the first week of treatment. (Childs et al., 2005). To assess the pain intensity for low back pain, the data measured at admission and at discharge were used.

2. Korean version of Oswestry Disability Index (K-ODI)

The Oswestry Disability Index (ODI) is composed of 10 items such as pain intensity, personal care, lifting, walking, sitting, standing, sleeping, sex life (if applicable), social life, and travel. Each item is checked on a scale of 0–five according to the patient status and the total score is converted to a percentage according to the number of response items. (Roland and Fairbank, 2000). The MCID for ODI has been reported to vary from 2.92 to 15.36 depending on the calculation method. (Copay et al., 2008). To assess functional disability of low back pain, the data measured at admission and at discharge were used. In this study, we used the validated Korean Version of the Oswestry Disability Index questionnaire. (Jeon et al., 2006).

3. Korean version of the Roland-Morris Disability Questionnaire (K-RMDQ)

The Roland-Morris Disability Questionnaire (RMDQ) consists of 24-item questions, and each question is checked regarding the patient’s different conditions. A high score signifies a greater degree of disability. (Roland and Fairbank, 2000). The MCID for RMDQ is considered to be a 30% change in the score when it is less than seven points and three points when it is above seven points at initial assessment. (Jordan et al., 2006). To assess functional disability of low back pain, the data measured at admission and at discharge were used. In this study, we used the validated Korean Version of the Roland–Morris Disability Questionnaire. (Lee et al., 2011).

4. Adverse events

All adverse events related to botanical drugs during the hospitalization period were described, and the severity and causality were evaluated. The severity of adverse events was assessed based on the Common Terminology Criteria for AEs (CTCAEs) scale, (National Cancer Institute, 2017), and causality assessment was conducted in accordance with the World Health Organization-Uppsala Monitoring Centre (WHO-UMC) causality assessment system. (World Health Organization (WHO)—Uppsala Monitoring Centre, 2018).

### Interventions

1. Acupuncture and Pharmacopuncture

Acupuncture treatment was performed by using disposable, sterilized stainless steel needles (0.25 × 30 mm, Dong-bang acupuncture, Boryeong, Korea) twice a day. Points were selected on the low back region such as *Shenshu* (BL23,腎兪), *Zhishi* (BL52, 志室), *Yaoyangquan* (GV3, 腰陽關), *Dachangshu* (BL25, 大腸兪) and were stimulated to a depth of 10–20 ㎜ for 15 min.

For pharmacopuncture treatment, A2-JS manufactured in Jaseng Wonoe Tangjunwon (Namyangju, Korea) was used (Product name; Jungsongouhyul, components; hGMP *Paeoniae Radix* (赤芍藥), hGMP *Salviae Miltiorrhizae Radix* (丹參), hGMP *Persicae Semen* (桃仁), hGMP *Myrrha* (沒藥), hGMP *Corydalis Tuber* (玄胡索), hGMP *Olibanum* (乳香), hGMP *Sappan Lignum* (蘇木), hGMP *Gardeniae Fructus* (梔子)). A total of 1cc dose was injected into five to six points of acupoints in the lumbar region using disposable insulin syringes (Almo-Erzeugnisse Erwin Busch GmbH, Germany) once a day.

2. Cupping therapy

Dry cupping and wet cupping were alternately performed. Dry cupping therapy was performed on the acupoints around the lumbar spine, and wet cupping was conducted by puncturing the acupoints around the lumbar region several times with a lancet and applying negative pressure using disposable, sterile cups (Dong-bang acupuncture, Boryeong, Korea) for 5 min.

3. Botanical drugs (Herbal medicine)

The meaning of “Chung-Pung” is as follows. The Chinese word for clean is chung (清). And Pung (風) is the Chinese word for wind, which is known as pathogenic wind. Pathogenic wind is characterized by rapid movement, swift changes, and ascending and opening actions. Chung-pung, then, refers to the ability to block out rapidly shifting pain patterns. The patients were administered Chung-Pung Decoction twice a day to activate blood and resolve stasis, relieve pain, relax sinews and activate collaterals during the hospitalization period. The patients took one pack at a time, and the capacity of one pack was 120 ml (120 ml * 2 times/day). A total of 40 packs are extracted at once using boiled water (120 ml per pack). About 4800 ml * 1.5 = 7200 ml of water was initially used for the extraction of boiled water. (Table 1).

4. Tuina manual therapy

**TABLE 1:** Composition and dose of one pack of Chung-Pung Decoction (1pack 120 ml).

Botanical drug name	Chinese Name	Amounts per Pack (g)
Alisma plantago-aquatica subsp. orientale (Sam.) Sam. [Alismataceae; Alismatis Rhizoma]	澤瀉	10
*Lonicera japonica* Thunberg. [Caprifoliaceae; Lonicerae Flos]	金銀花	6
Atractylodes japonica Koidz. [Compositae; Atractylodis Rhizoma Alba et rhizome]	白朮	6
Polyporus umbellatus (Pers.) Fr. [Polyporaceae; Polyporus]	猪苓	6
Poria cocos Wolf. [Polyporaceae; Poria Sclerotium]	茯苓	6
Ulmus macrocarpa Hance. [Ulmaceae; Ulmi Cortex]	楡白皮	4
*Taraxacum* platycarpum H. Dahlstedt. [Compositae; Taraxaci Herba]	蒲公英	4
*Glycyrrhiza* uralensis Fischer. [Leguminosae; Glycyrrhizae Radix et Rhizoma]	甘草	2
Cinnamomum cassia J.Presl [Lauraceae; Cinnamomi Cortex]	肉桂	2

The patients took one pack at a time, and the capacity of one pack was 120 ml (120 ml * 2 times/day). A total of 40 packs are extracted at once using boiled water (120 ml per pack). About 4800 ml * 1.5 = 7200 ml of water was initially used for the extraction of boiled water.

Tuina manual therapy was conducted once a day in a prone position, including extension and rearrangement of intervertebral joints in the lumbar region and muscle compression technique around the lumbar spine. (Korean Society of Chuna Manual Medicine for Spine & Nerves, 2017).

5. Physical therapy

Infrared therapy (IR), transcutaneous electrical nerve stimulation (TENS), ultra-sound treatments were applied to the lumbar region once a day.

### Statistical analysis

In this study, statistical analysis was performed using Windows SPSS version 12.0 (IBM Corp., Armonk, NY, United States). Demographic characteristics were described using frequency and percentages in categorical data and using mean, standard deviation and percentile in continuous data. Differences between the outcome variables at admission and discharge were analyzed by a paired *t*-test. The correlation between hospitalization period and symptom improvement was analyzed by Pearson’s correlation analysis. The test was two-tailed and a *p*-value of less than 0.05 was considered statistically significant. There were no missing data.

### Ethical review

This study met all criteria to qualify as exempt from the institutional review board (IRB) and exemption was approved by Chung-Yeon Korean Medicine Hospital IRB (IRB No. CY-IRB 2018-09-002).

## Results

### Patient characteristics

**FIGURE 1:**
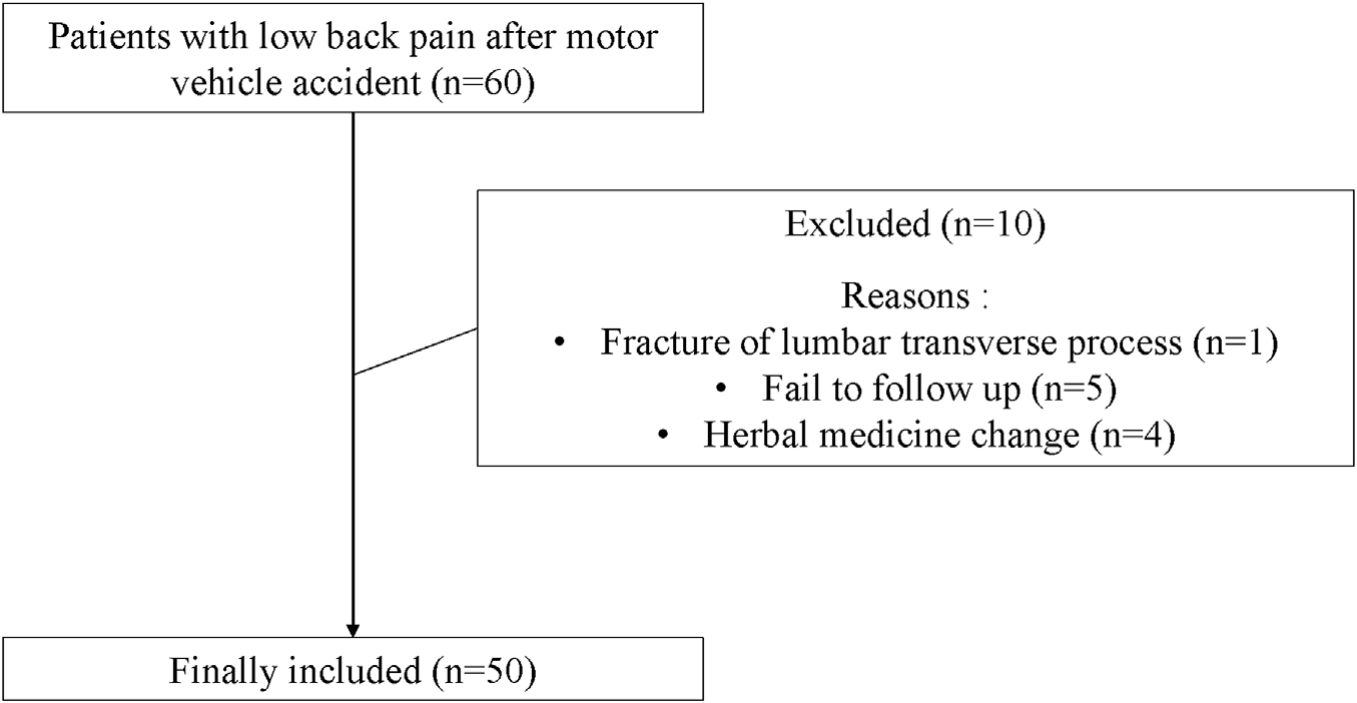
Flow chart.

A total of sixty patients were treated with comprehensive KM treatment during this period. Five cases failed to follow-up due to sudden discharge (follow up outcome was not measured), one case of low back pain was due to fracture of the transverse process of lumbar vertebra, four patients had changes in botanical drugs prescription due to alteration of the chief complaint or adverse events. Therefore, a total of 10 cases were excluded. We reviewed 50 cases that were included in this study retrospectively based on the electronic medical record (Figure 1).

30 males and 20 females were included. The mean age of patients was 40.72 ± 13.31 years and the mean days of hospitalization period were 7.22 ± 3.84 days (Table 2).

**TABLE 2:** Demographic Characteristics of Subjects.

Variables	Frequency or Mean	Minimum	Percentiles	Maximum		
			25%	Median	75%	
Gender						
Male	30 (60%)					
Female	20 (40%)					
Past history and Medication	cardiovascular disease; 9 (18%), diabetes mellitus; 4 (8%), lumbar disc surgery; 4 (8%), appendicitis operation; 5 (10%), analgesics or muscle relaxants; 3 (6%)					
Age (years)	40.72 ± 13.31	20	29	38.5	52.25	66
Height (cm)	168.58 ± 9.09	148	162.75	170	176.25	183
Weight (kg)	67.88 ± 16.96	41	56	65	76.5	120
Period from onset day (days)	3.62 ± 2.02	1	2	3	5	11
Hospitalization period (days)	7.22 ± 3.84	2	4	5.5	10.25	16

Categorical data are expressed with frequency (ratio). Continuous data are expressed with mean ± standard deviation.

### Changes in VNRS, K-ODI and K-RMDQ values

**FIGURE 2:**
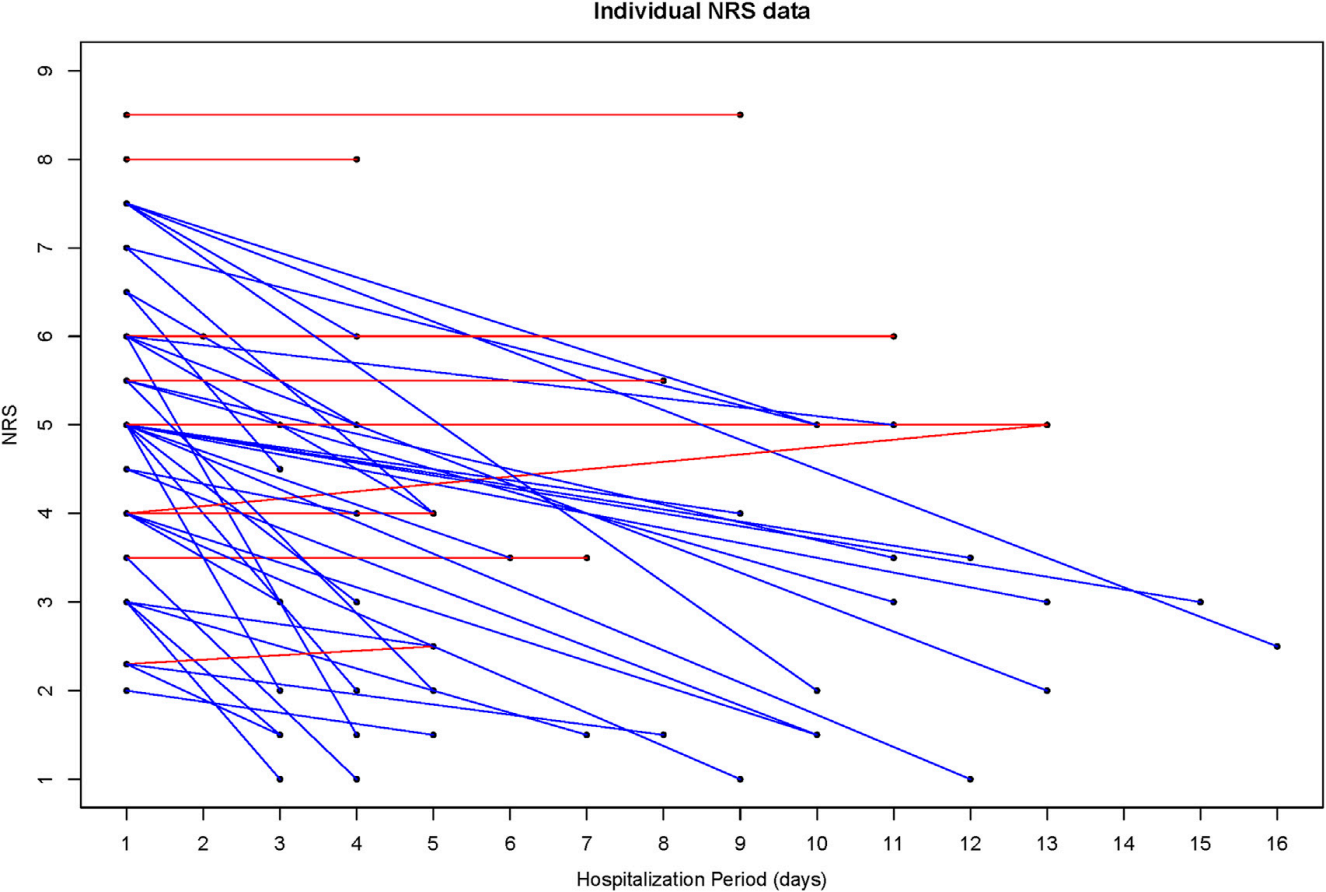
Individual data of the numeric rating scale (NRS).

**FIGURE 3:**
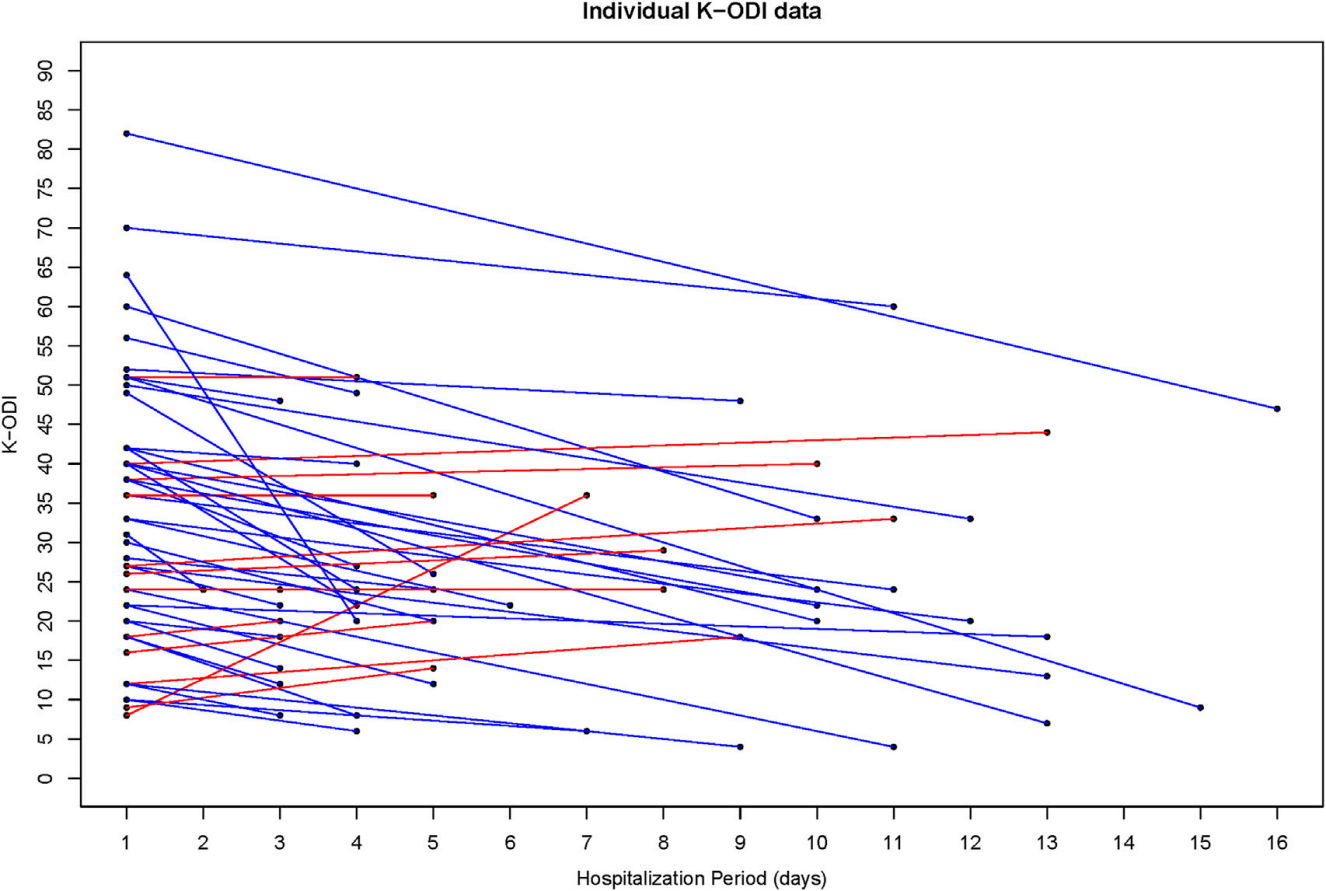
Individual Data of the Korean version of the Oswestry Disability Index (K-ODI).

**FIGURE 4:**
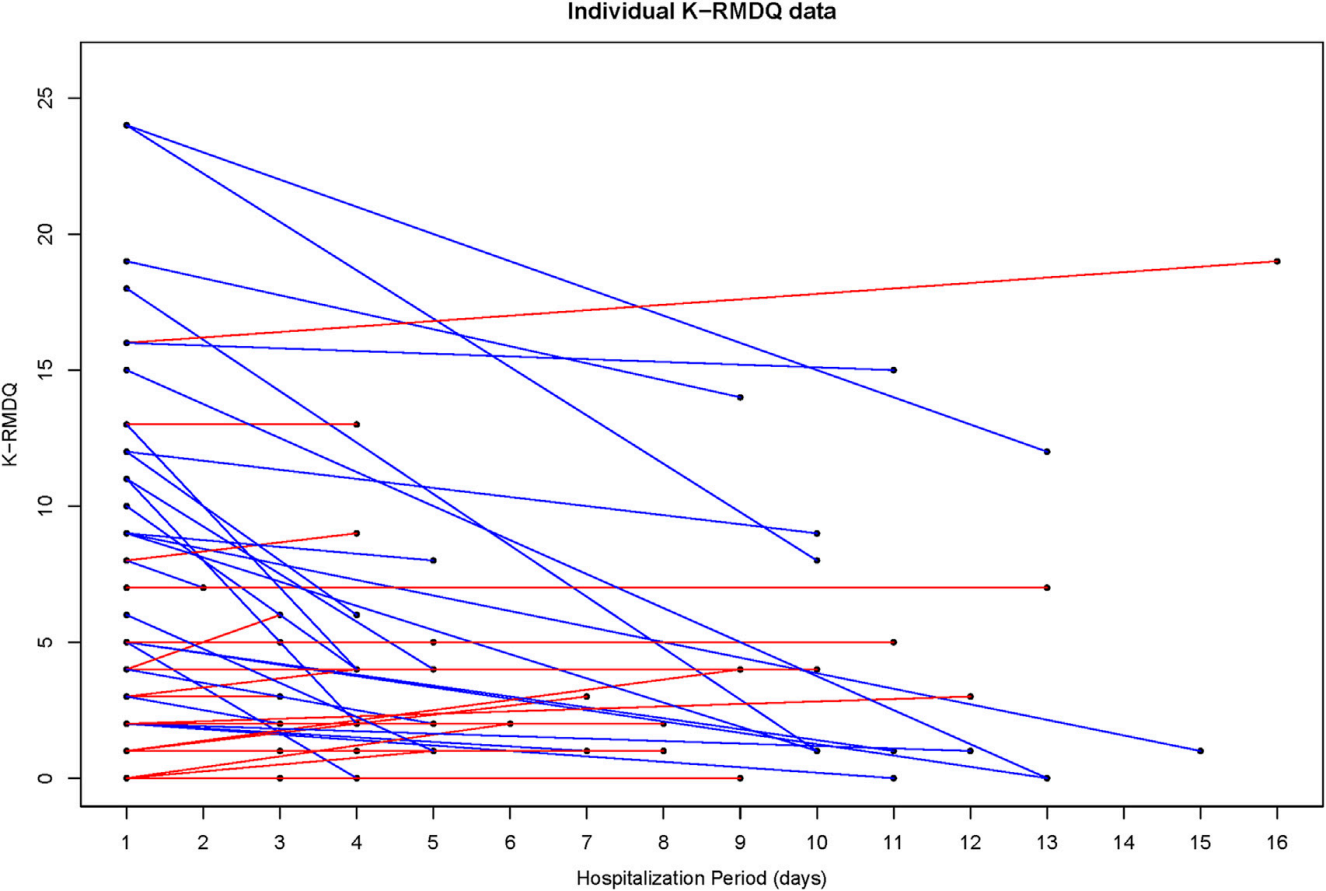
Individual Data of the Korean version of the Roland-Morris Disability Questionnaire (K-RMDQ).

VNRS values decreased from 5.06 ± 1.60 at admission to 3.40 ± 1.81 at discharge. K-ODI values decreased from 33.38 ± 16.88 at admission to 24.54 ± 13.63 at discharge. Also, there was a decrease in K-RMDQ from 6.84 ± 6.27 at admission to 4.14 ± 4.38 at discharge. VNRS, K-ODI, and K-RMDQ were significantly improved before and after the integrative Korean Medicine treatment package for low back pain including Chung-Pung Decoction (Table 3). Individual data of VNRS, K-ODI and K-RMDQ are presented in Figures 2–4 respectively.

**TABLE 3:** Change of VNRS, K-ODI, K-RMDQ value (N = 50).

	Admission	Discharge	Change value [95% CI]	*p*-value
VNRS	5.06 ± 1.60	3.40 ± 1.81	1.66 ± 1.46 [1.26, 2.06]	<0.001*
K-ODI	33.38 ± 16.88	24.54 ± 13.63	8.84 ± 12.90 [5.26, 12.42]	<0.001*
K-RMDQ	6.84 ± 6.27	4.14 ± 4.38	2.70 ± 4.81 [1.37, 4.03]	<0.001*

95% CI, indicates 95% Confidence interval. We presented upper and lower 95% CI.

VNRS, the Verbal Numerical Rating Scale; K-ODI, the Korean version of the Oswestry Disability Index; K-RMDQ, the Korean version of the Roland-Morris Disability Questionnaire. *p*-value was derived from paired *t*-test.

Change value means the value of each variable on admission minus value of each variable on discharge. We also provide lower and upper 95% confidence interval with mean ± standard deviations.

Data are presented with mean ± standard deviation. *:*p* < 0.05.

The blue line improved individual patients; the red line worsened individual patients.

The blue line improved individual patients; the red line worsened individual patients.

The blue line improved individual patients; the red line worsened individual patients.

There was no significant difference in the correlation analysis between the hospitalization period and improvement of VNRS, K-ODI and K-RMDQ value (data not shown).

### Adverse events

Two adverse events were reported out of a total of 60 cases in which Chung-Pung Decoction was taken from 1 July 2018 to 30 September 2018. One was abdominal pain lasting 2 to 3 h, and the other was abdominal distension after botanical drugs administration. In the case of a patient who complain of abdominal pain, severity was assessed for Grade 1 (Mild) based on CTCAEs scale and causality was evaluated as Probable/Likely causality in accordance with WHO-UMC causality assessment system. Abdominal distension was assessed as Grade 1 severity and Probable/Likely causality when the same criteria were applied.

## Discussion

In this study, 50 patients with acute low back pain after MVAs were treated with comprehensive KM treatment which included Chung-Pung Decoction, acupuncture, pharmacopuncture, cupping therapy and tuina manual therapy. We analyzed the pain intensity and functional disability scales associated with the low back region before and after treatment and adverse events during the treatment period and confirmed effectiveness and safety based on real-world data. The results of this study showed that the pain intensity score, VNRS, improved by 1.66 points from 5.06 ± 1.60 to 3.40 ± 1.81. Considering that MCID of for low back pain was 1.5 points at the first week of treatment, (Childs et al., 2005), pain intensity had improved clinically as well as statistically. The RMDQ values also improved by 2.7 points from 6.84 ± 6.27 to 4.14 ± 4.38 in this study, and this is consistent with previous studies in which MCID of the RMDQ is considered to be a 30% change in the score when it is less than seven points. (Jordan et al., 2006). Therefore, the degree of functional disability also improved clinically and statistically. K-ODI improved 8.84 ±12.90 points which also means disability improved clinically and statistically. In addition, since the incidence of adverse events was low as 3.3% and severe adverse events did not occur, comprehensive KM treatment including Chung-Pung Decoction can be deemed a relatively safe treatment. Overall, comprehensive KM short-term treatment for early low back pain patients due to MVAs is considered to be an effective and safe treatment strategy.

As a conventional treatment for LBP after MVAs, medications such as acetaminophen and muscle relaxant are used including NSAIDs. (Wong et al., 2017). However, limitations exist as each drug can have side effects. Muscle relaxants can cause sleepiness and dizziness and NSAIDs can make side effects such as abdominal discomfort, diarrhea, and gastrointestinal bleeding if taken for a long period of time. (Qaseem et al., 2017). In addition, in the case of oral NSAIDs, one study has shown that there is controversy over their effectiveness compared to placebo when applied to recent-onset LBP. (Wong et al., 2016).

To compensate for the limitations and effectiveness of these conventional therapies, KM treatment can be proposed as a promising alternative treatment. In Korea, several case studies and clinical studies have reported on the effectiveness of KM treatment for the sequelae of MVAs, (Park et al., 2015; Han et al., 2018), and it was found that the satisfaction of the MVA patients treated with KM treatment was very high. (Kim et al., 2018). However, not many studies looked into real-world data on treatment packages that include specific botanical drugs prescriptions.

Chung-Pung Decoction used in this study is a unique botanical drug (herbal medicine) prescription that is frequently used in KM hospitals. Botanical drugs consist of *Alismatis Rhizoma*, *Lonicerae Flos*, *Atractylodis Rhizoma Alba*, *Polyporus*, *Poria Sclerotium*, *Ulmi Cortex*, *Taraxaci Herba*, *Glycyrrhizae Radix et Rhizoma* and *Cinnamomi Cortex*. It is prescriped for relieving various musculoskeletal pain and disc disorders. In the case of *Alismatis Rhizoma*, the ethanol extract has been shown to reduce NF-kB activity and the gene expression associated with and interleukin (IL) -1β, iNOS, and COX-2. (Han et al., 2013). *Lonicerae Flos*, which is used for various inflammatory diseases, has also been shown to significantly reduce NO and PGE2 production by its hot aqueous extract and ethanol extract for lipopolysaccharide-stimulated macrophages. (Yun and Lee, 2012). In addition, luteolin, derived from *Lonicerae Flos*, has been shown to significantly reduce the induction of various inflammatory cytokines such as interleukin (IL)-8, IL-6 and necrosis factor (TNF)-α. (Kang et al., 2010). It has been found that the extract solution of *Ulmi Cortex* significantly reduces the production of IL-2 and TNF-α in peritoneal macrophages and synovial membranes for arthritis animal models, (Song et al., 2007), and *Cinnamomi Cortex* pharmacopuncture has anti-inflammatory effects such as inhibition of ERK1/2 phosphorylation and COX-2, iNOS expression. (Kim and Roh, 2012). It is assumed that the complex action of the above-mentioned anti-inflammatory botanical drugs may have been effective in relieving acute lumbar inflammation.

In addition, acupuncture combined with this treatment is known to have analgesic actions by locally lowering the levels of inflammatory mediators and enhancing the descending inhibitory effect. (Lai et al., 2019). A study has shown that pain relief effects of wet cupping therapy can be mediated by increasing the expression of heat shock protein 70 (HSP70) and ß-endorphin. (Subadi et al., 2017). Tuina manual therapy is performed on the joints and soft tissues of the body, and it is known to move and rearrange the joints and to have an effect on stretch function and pain relief of the soft tissues. (Korean Society of Chuna Manual Medicine for Spine & Nerves, 2017). A systematic literature review suggested that Tuina-focused integrative Chinese Medicine therapy can be effective in improving the pain and functional conditions in low back region. (Kong et al., 2012).

The strength of this retrospective chart review is that it looked into the synergistic effect of integrative KM treatment in 50 cases of LBP patients, with individual data visualization based on real-world practice using various clinical outcomes. In addition, unlike previous KM case studies, the subjects of this study took the same botanical drugs and was treated using standardized treatment strategies for acupuncture, pharmacopuncture, tuina manual therapy, moxibustion, cupping therapy and physical therapy, and this provides data on standard treatment strategies rather than individualized treatments. Even more, within a relatively short period of 7 days of hospitalization, this study showed that comprehensive KM treatment could clinically and significantly lower LBP. The incidence of adverse events during the treatment period was low and severity was mild, showing possibility of using alternative treatment as a safe, primary treatment for LBP patients after MVAs. More importantly, most of the previous studies were focused on cervical pain, and this study provides significant clinical data and insight on LBP which is the second most common complaint after cervical pain.

Nevertheless, this study does have some limitations. First, the efficacy of comprehensive KM cannot be guaranteed in the absence of comparative data to untreated patients, physical therapy, or spontaneous recovery. Change values in VNRS, K-ODI, and K-RMDQ should be contrasted with the control group. Moreover, historical control group data was not sufficient, so it was difficult to compare with existing studies. A controlled group clinical design study is needed. Also, patients’ compliance and satisfaction with botanical drugs were not assessed. Second, Although the incidence and severity of adverse events was reported to be low and mild, safety evaluation was not corroborated through blood tests. Blood tests for MVA patients using KM comprehensive treatment are crucial to determining the cause of adverse events in further clinical practice and clinical research. However, the rate of hepatic injury due to botanical drugs is estimated to be very low as a recent multi-institutional study suggested that the incidence rate of herb-induced hepatic injury is known to be about 0.6% in Korea. (Cho et al., 2017). In addition, since this study looked into the comprehensive treatment effects of a KM treatment package, it was not able to further discern which of the treatments had greater effects on the improvement of LBP that occurred after MVAs. However, it is also significant to look into the synergetic effects of an integrated package as a whole, which most accurately reflects the clinical practice setting. Our study was unable to quantify the benefit of comprehensive KM treatment over single-KM therapy. Therefore, using factorial design research, it is also necessary to confirm the synergistic effect and effectiveness of each particular intervention in follow-up studies. Another limitation of this study is that it did not evaluate the long-term effects due to different hospitalization periods of each patient and difficulty in clinical follow up. In the future, prospective, practical, observational studies are needed to complement the above limitations, including long-term follow-up, and it should also be accompanied by a qualitative study to obtain a more detailed patient perspective.

## Conclusion

Comprehensive KM treatment was provided for MVA-induced LBP patients. Pain intensity (VNRS) and functional disability status (K-ODI and K-RMDQ) significantly improved. Short-term KM treatment package might be a safe and effective treatment strategy to alleviate acute stage pain resulting from a car accident. However, further prospective and controlled clinical research is warranted.

## Data Availability

The raw data supporting the conclusions of this article will be made available by the authors, without undue reservation.
